# Characterizing the Cracking Behavior of Large-Scale Multi-Layered Reinforced Concrete Beams by Acoustic Emission Analysis

**DOI:** 10.3390/s25123741

**Published:** 2025-06-15

**Authors:** Yara A. Zaki, Ahmed A. Abouhussien, Assem A. A. Hassan

**Affiliations:** 1 Department of Civil Engineering, Faculty of Engineering and Applied Science, Memorial University of Newfoundland, St. John’s, NL A1B 3X5, Canada; azakielsayed@mun.ca (Y.A.Z.); ahassan@mun.ca (A.A.A.H.); 2 Kinectrics, 393 University Ave, Toronto, ON M5G 1E6, Canada

**Keywords:** rubberized engineered cementitious composites, acoustic emission analysis, first crack detection, damage quantification, sensor location

## Abstract

In this study, acoustic emission (AE) analysis was carried out to evaluate and quantify the cracking behavior of large-scale multi-layered reinforced concrete beams under flexural tests. Four normal concrete beams were repaired by adding a layer of crumb rubberized engineered cementitious composites (CRECCs) or powder rubberized engineered cementitious composites (PRECCs), in either the tension or compression zone of the beam. Additional three unrepaired control beams, fully cast with either normal concrete, CRECCs, or PRECCs, were tested for comparison. Flexural tests were performed on all the tested beams in conjunction with AE monitoring until failure. AE raw data obtained from the flexural testing was filtered and then analyzed to detect and assess the cracking behavior of all the tested beams. A variety of AE parameters, including number of hits and cumulative signal strength, were utilized to study the crack propagation throughout the testing. Furthermore, *b*-value and intensity analyses were implemented and yielded additional parameters called *b*-value, historic index [*H (t)*], and severity (*S_r_*)_._ The analysis of the changes in the AE parameters allowed the identification of the first crack in all tested beams. Moreover, varying the rubber particle size (crumb rubber or powder rubber), repair layer location, or AE sensor location showed a significant impact on the number of hits and signal amplitude. Finally, by using the results of the study, it was possible to develop a damage quantification chart that can identify different damage stages (first crack and ultimate load) related to the intensity analysis parameters (*H (t)* and *S_r_*).

## 1. Introduction

Concrete structures, including bridges, parking garages, offshore structures, and other marine structures, are exposed to external factors such as harsh environmental conditions, impact stresses, and high mechanical loading. Exposure to such factors eventually leads to deterioration of the concrete structures, which immediately affects the intended service life [1]. Repair or strengthening of those deteriorated structures is, therefore, inevitable to improve the functionality and performance of such structures [1,2]. Recently, engineered cementitious composites (ECCs) were introduced for use in repairing concrete structures due to their high performance characteristics compared to common cementitious repair materials [3]. ECCs exhibit very high ductility, with strain-hardening and multi-cracking behavior.

ECCs contribute to a tensile strain capacity that varies from 3% to 7% when compared to normal concrete, which exhibits a tensile strain capacity of 0.01% [4]. Since ECCs have strain-hardening characteristics, their cracks are characterized by fiber-bridging mechanisms and tight width [5,6]. ECCs generally comprise relatively high volumes of fibers such as Polyvinyl fibers (PVA) and high volumes of fine silica sand [3]. The volume of PVA fibers in ECCs is normally 2% [7]. Recently, waste rubber started to be viewed as an economically recyclable material for use in the production of ECCs [8]. When rubber aggregates are placed in ECCs, the strain rate and toughness tend to increase [9]. Furthermore, rubberized concrete exhibits higher strain rates, which allows for greater deformation and contributes to higher ductility compared to normal concrete [10,11,12,13]. Previous studies indicated that increasing the amount of rubber aggregates in concrete decreased the compressive and tensile strengths. This is due to the difference between the modulus of elasticity of rubber particles and cement paste [3,14]. However, the negative effect of rubber on the strength is alleviated in ECC mixtures because of the presence of a high volume of fibers. The inclusion of PVA fibers in ECCs not only helps to alleviate the reduction in mechanical strength, but also improves the ductility, energy absorption, and impact resistance of concrete [1].

Acoustic Emission (AE) monitoring is a non-destructive method used to monitor the structural health of many existing concrete structures [15,16,17,18]. The AE technique has the ability to detect the strain energy in a structure by monitoring the released elastic waves. The strain energy is recorded by AE sensors and related to different types of damage that are occurring in the monitored structure [19]. AE analysis also has the ability to categorize different types of failures and to quantify the severity of damage [20].

AE analyses have shown their effectiveness in evaluating the cracking and damage behavior of self-compacting rubberized concrete [21,22], fiber-reinforced concrete [23,24,25,26], and strain-hardening cement-based composites [27,28]. In particular, *b*-value analysis (amplitude/number of hits) and intensity analysis (historic index *H (t)* and severity *S_r_*) were found to be very useful tools in assessing the cracking behavior (micro-cracking and macro-cracking) of composite structures [29]. In addition, RA (rise time/amplitude) vs. average frequency (AF) analysis was also utilized as a good crack classification tool to differentiate between different types of failure modes (shear, tensile, or debonding). Ranjbar et al. used RA and AF analysis to categorize the types of failure of multi-layered composite beams consisting of geopolymer, CFRP, FRCM, and normal concrete [30,31,32].

To date, the implementation of AE analyses for evaluating/quantifying the cracking behavior of multi-layered beams is limited. There is a need to study the change in the AE parameters in multi-layered composite beams due to the variation in the wave propagation characteristics through the two layers, especially when rubberized engineering cementitious composites (RECCs) are used. Furthermore, the location of the sensor with respect to the two different layers is anticipated to be a factor, owing to the contribution of rubber particles to sound absorption and the properties of interfacial bond between the two layers. In addition, the impact of using multi-layered RECCs on the effectiveness of *b*-value and intensity analyses for the purpose of detection/quantification of the cracking and severity of damage requires further investigation. This study aims to assess the effectiveness of *b*-value and intensity analysis for characterizing the crack propagation in RECC beams with different strengthening locations and different rubber particle sizes. This study also attempts to quantify the cracking behavior of multi-layered RECC beams by developing a damage classification tool for different cracking stages (first crack and ultimate load) with the aid of intensity analysis parameters. The effect of using different strengthening locations in multi-layered RECC beams on AE parameters is also examined in this study.

## 2. Research Significance

The implementation of AE monitoring and analyses has proven its efficacy in damage classification, including detection of crack propagation in several concrete and composite structures. However, there is a gap in the literature regarding the application of AE analyses in multi-layered RECC beams, especially with variable rubber particle sizes. The integrity of AE analyses in evaluating the structural performance of RECC composite beams requires further investigation, as rubber particles have sound-absorbing properties, which may have a significant impact on the AE parameters. Alternating the RECC layer along with the strengthening/sensor location is anticipated to show prominent changes on the AE signal parameters. Moreover, AE wave propagation is expected to be different in repaired beams when compared to normal concrete beams due to signal attenuation. The literature also lacks information regarding the implementation of *b*-value and intensity analyses in the crack quantification of multi-layered RECC beams. The AE analyses presented in this paper will aid in categorizing the different damage stages and will help to better understand the cracking behavior of multi-layered RECC beams (in terms of crack initiation and crack propagation).

## 3. Testing Program

### 3.1. Material Properties

**Figure 1:**
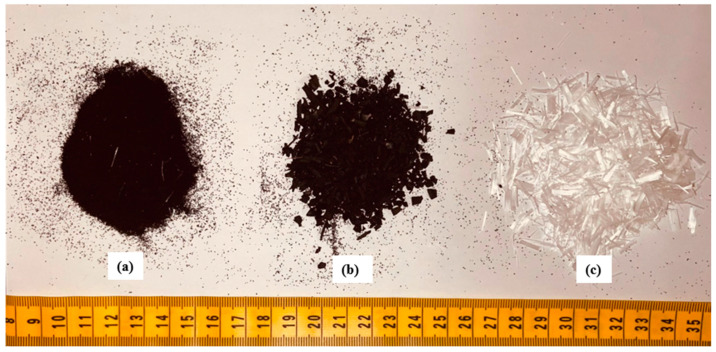
(**a**) Powder rubber; (**b**) crumb rubber; (**c**) PVA fibers.

Table 1 represents the three mixtures used in this study (one normal concrete mixture and two rubberized engineering cementitious composite mixtures). The normal concrete mixture (NC) consisted of GU type Portland cement, complying with ASTM C150 Type 1 [33]. The NC mixture also consisted of coarse aggregate (natural crushed stone), with a maximum aggregate size of 10 mm, and fine aggregate (natural sand). The coarse and fine aggregate had a specific gravity of 2.6 and an absorption ratio of 1%. RECC mixtures consisted of GU type Portland cement, complying with ASTM C150 Type 1 [33], and fly ash (FA), complying with ASTM C618 Type F [34]. The fine aggregate used for RECC mixtures is silica sand, with a maximum grain size of 0.4 mm and a specific gravity of 2.65. Crumb rubber (CR) and powder rubber (PR) were used to partially replace the silica sand. Both CR and PR had a specific gravity of 0.95 and 0.86, respectively, with negligible water absorption. The CR has a particle size that ranges from 0.425 mm to 4.75 mm, while the PR particle size is 0.425 mm. The replacement level for both types of rubber was 20% (by volume). This replacement was determined based on a previous study [14] focused on optimizing the content of CR and PR in RECCs. PVA fibers (2% by volume) were used in all RECC mixtures. The PVA fibers had a length of 8 mm, tensile strength of 1600 MPa, modulus of elasticity of 40 GPa, and a specific gravity of 1.3 (Figure 1). More details of the mix design can be found elsewhere [1].

### 3.2. Selection of Beam Specimens

The seven tested beams were selected and as follows:

- One NC beam was fully cast with a normal concrete mixture and was used as a control beam/mixture for comparison (denoted as B1).
- Two RECC beams were fully cast with ECCs containing different rubber sizes (CR or PR). B2 consisted of CR, while B3 consisted of PR. These beams were referred to as CRECC and PRECC, respectively.
- Two beams repaired with a top RECC layer in the compression zone (B4 and B5) contained different sizes of rubber (CR or PR). B4 contained an RECC layer of CR, while B5 contained an RECC layer of PR. These beams had an NC layer with a depth of 165 mm in the tension zone and RECC layers (either with CR or PR) with a depth of 85 mm in the compression zone. Both beams were donated as CRECC-C and PRECC-C, respectively. The NC layer with a depth of 165 mm was poured first. Following the initial setting, the top surface of the NC layer was roughened to provide a good bonding with the new RECC layer. After one day of curing, the NC surface was cleaned with air pressure to remove any loose particles, and the new RECC layer was poured with a depth of 85 mm in the compression side.
- Two beams repaired with a bottom RECC layer in the tension zone (B6 and B7) contained different sizes of rubber (CR or PR). B6 contained an RECC layer of CR, while B7 contained an RECC layer of PR. These beams had RECC layers with a depth of 85 mm in the tension zone and an NC layer with a depth of 165 mm in the compression zone and were designated as CRECC-T and PRECC-T, respectively. The NC layer with a depth of 165 mm was poured first (the beam was poured upside down in the case of RECC in the tension side). Following the initial setting, the top surface of the NC layer was roughened to provide a good bonding with the new RECC layer. After one day of curing, the NC surface was cleaned with air pressure to remove any loose particles, and the new RECC layer was poured with a depth of 85 mm in the top side (tension side).

### 3.3. Flexural Loading Test Setup and Loading Procedure

**Figure 2:**
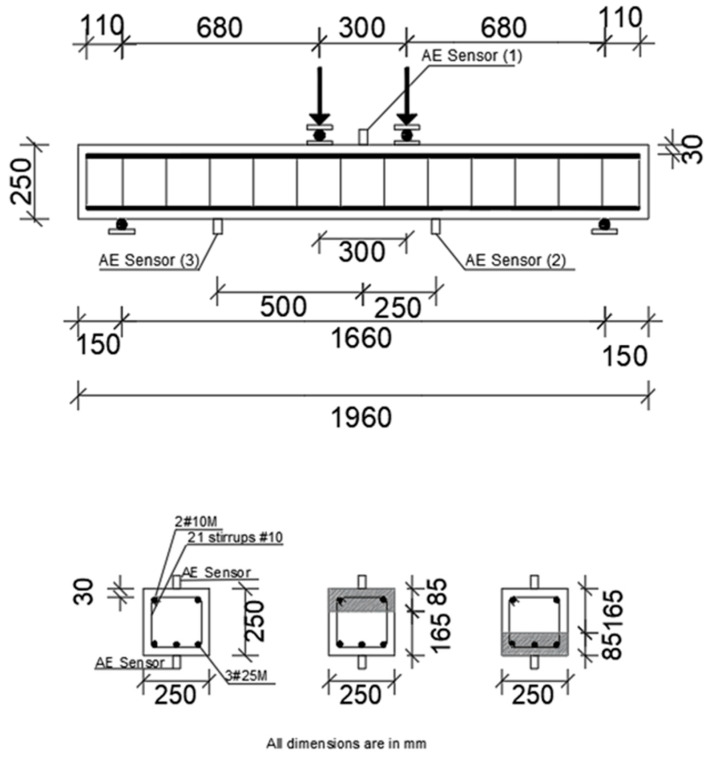
Test setup for all tested beams.

Dimensions, steel reinforcement, and flexural test setup for all beams are illustrated in Figure 2. The cross-sectional dimensions for all tested beams were 250 mm × 250 mm. All beams had a total length of 1960 mm and an effective load span of 1660 mm. Three 25 M bars were used in the tension zone, while two 20 M bars were used in the compression zone. The shear reinforcement was 10 mm diameter stirrups with a spacing of 100 mm. The concrete cover for the tested beams was 30 mm. All beams were tested under four-point loading and simply supported with a span of 1660 mm (Figure 2). The single-point load on a steel beam was applied using an actuator with a load capacity of 500 kN. The load was distributed on two points spaced at 300 mm. The load was first applied until the first crack occurred and then applied gradually using a displacement control rate in increments until failure. After each load increment, the cracks were measured using a crack microscope. Table 2 illustrates the results of the flexural testing of all beams.

## 4. AE Monitoring Procedure

### 4.1. AE Setup

Three sensors at different locations were attached to each beam to record the released AE during the testing. As shown in Figure 2, sensor 1 was placed in the mid-span of the beam, sensor 2 was placed at a distance of 250 mm measured from sensor 1, and sensor 3 was placed at a distance of 500 mm measured from sensor 1. All AE sensors were attached to the surface of the beam using a two-part epoxy adhesive prior to the flexural tests. The AE sensors used in this study were piezoelectric sensors with an integral preamplifier having a model number of R61-AST [35]. The recording and processing of the signals released during the testing were carried out by an AE system and an AE signal processing software (AEwin for USBTM software, Version E3.32) from Mistras Group [36]. An amplitude threshold of 40 dB was kept constant during the testing. Other data acquisition parameters used to set up the hardware are illustrated in Table 3. Parameters such as amplitude, signal strength, duration, energy, absolute energy, counts, rise time, average frequency, and peak frequency were obtained. The definitions of the previously mentioned AE terms are found in ASTM E1316 [37].

### 4.2. Post-Testing AE Data Filtering

An amplitude-duration-based filter (Swansong II filter) was used to filter all the raw data extracted from the four-point load tests. The filtering process is carried out in order to minimize noise/unwanted wave reflections that may occur in the beams [38]. This amplitude-duration filter was successfully used in several previous investigations in AE monitoring in the concrete industry [39,40,41]. The filter works by assuming that the real AE events with high values of amplitude are characterized by long magnitudes of duration, and vice versa [42]. The rejection limits for the AE signals are presented in Table 4. After completing the filtering process, the remaining AE signals are considered to be real AE sources resulting from the crack initiation and propagation from the four-point load tests.

## 5. AE Analysis and Processing

### 5.1. b-Value Analysis

A variety of AE parameters were analyzed in this paper to classify the cracking behavior in all beams. Firstly, traditional AE parameters that were directly extracted from the AE monitoring data, such as signal amplitude, number of hits, and cumulative signal strength (CSS), were evaluated. Secondly, *b*-value analysis was performed by calculating the *b*-value parameter from the amplitude and number of hits. The *b*-value was then used to indicate the severity of damage (crack development) in the tested beams. This analysis was originally used in the seismic analysis and was then further applied in AE analysis [43]. *b*-value analysis was successfully employed in several previous investigations in concrete materials and structures [40,44,45,46]. The *b*-value was calculated using Equation (1) for all tested beams.

(1) log *N* = *a* − *b* log *A*

where *N* = number of hits with an amplitude larger than *A*; *A* = signal amplitude (dB); *a* = empirically derived constant; and *b* = *b*-value [44,45,46]. The value of the constant ‘a’ was obtained by plotting log *N* (*y*-axis) versus log *A* (*x*-axis) for the seven beams. The average magnitude of ‘*a*’ for all beams was then obtained and applied in Equation (1).

### 5.2. Intensity Analysis

In addition to the *b*-value analysis, intensity analysis was also carried out for the tested beams. The intensity analysis was performed on the signal strength of the acquired AE signals and generated two additional parameters: historic index [*H (t)*] and severity [*S_r_*]. These parameters were utilized in several previous investigations to detect and quantify damage in concrete structures [41,42,47,48]. The historic index spots the locations where there are sudden changes in the CSS curve. *H (t)* was calculated for all the beams using the following Equation (2):

(2) $$H\left(t\right)=\frac{N}{N-K}\frac{\sum_{i=K+1}^{N}S_{oi}}{\sum_{i=1}^{N}S_{oi}}$$

where *N* = cumulative number of hits up to a certain time (*t*), while *S_oi_* is the signal strength of the *i*th signal.

The severity indicates the average value of signal strength of *J* number of hits and was calculated by Equation (3).

(3) $$S_{r}=\sum_{i=1}^{J}\frac{S_{oi}}{J}$$

The values of *K* and *J* in Equations (2) and (3) were kept as constants for all the seven beams. The values for the previously mentioned variables depend on the damage mechanism and the material being analyzed. For this study, the magnitudes for *K* and *J* were acquired from intensity analysis successfully implemented from similar previous studies [39,42].

The *K* in Equation (2) was calculated based on the cumulative number of hits (*N*) as follows:

(a) *K* = 0: if *N* ≤ 50, (b) *K* = *N* − 30: if 51 ≤ *N* ≤ 200, (c) *K* = 0.85 *N*: if 201 ≤ *N* ≤ 500, and

(d) *K* = *N* − 75: if *N* ≥ 501.

However, the value for *J* was kept at a constant of 50 for all tested beams based on the analysis conducted in similar previous studies [39,42].

## 6. Results and Discussion

The test results including the modes of failure, mid-span deflections, and number/width of cracks are illustrated in Table 2. All the control beams (PRECC, CRECC, and NC) failed in flexural mode, in which the longitudinal reinforcement in the tension zone yielded, and the concrete crushed in the compression zone of the beam. RECC control beams incorporating PR or CR exhibited higher deformation when compared to the NC beam (Table 2). The improvement in the deformation characteristics of the RECC beams when compared to NC was attributed to the nature of the rubber particles, which are characterized by large elastic deformations before failure [49]. RECC beams also showed a large number of cracks with tight widths compared to the NC beam. The RECC beam with CR (CRECC) displayed 39 cracks with a maximum width of 2 mm, whilst the RECC beam with powder rubber (PRECC) displayed 36 cracks with a maximum width of 2.2 mm (Table 2). The increase of the number of cracks in the RECC beams when compared to the NC beam was due to the presence of fibers, which tend to control cracks from expanding.

### 6.1. Crack Detection Using AE Analysis

**Figure 3:**
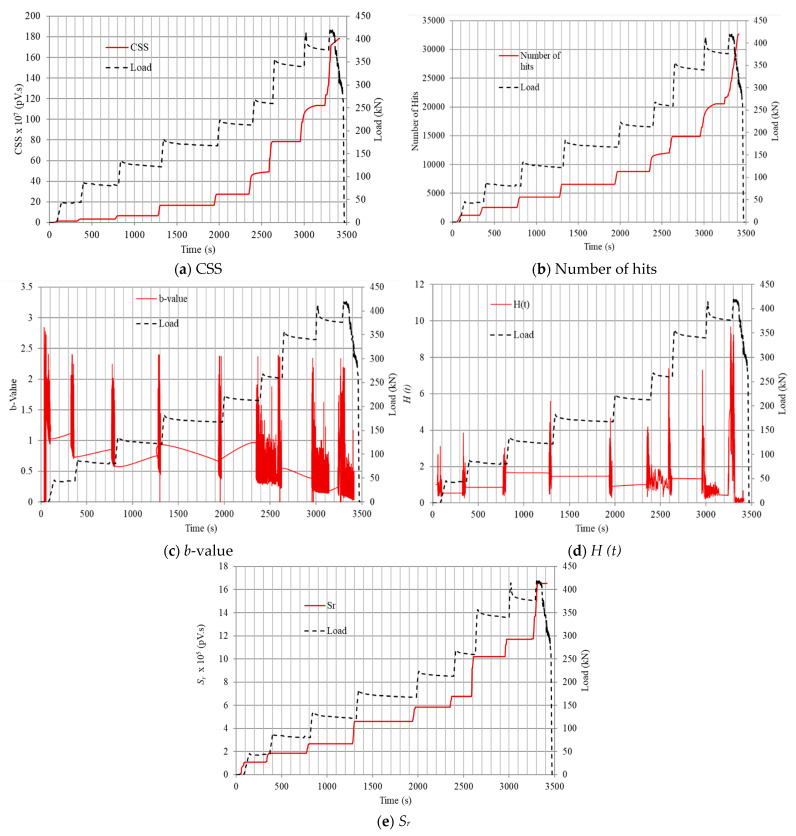
AE parameters and load vs. time curves for PRECC beam sensor 2: (**a**) CSS; (**b**) number of hits; (**c**) *b*-value; (**d**) *H (t)*; (**e**) *S_r_*.

In this section, the effect of the cracking behavior of the seven beams on the AE signals and parameters obtained during the testing process was studied. Figure 3 illustrates a number of AE parameters from the PRECC beam (sensor 2) that was chosen to be an example and representative of other beams and sensors. Several AE parameters such as CSS, number of hits, amplitude, *b*-value, *H (t),* and *S_r_* were analyzed over time for the PRECC beam in Figure 3. All the previously mentioned parameters were analyzed throughout the testing procedure until failure. In Figure 3a,c, both the CSS and number of hits showed very similar trends and experienced an overall increase over time. The increase in the AE activity is an indication of crack occurrence and propagation in the tested beam till failure. In particular, the first significant activity that can be correlated to the appearance of the first crack was detected at about 70 s (Figure 3). To further elaborate, the first change in slope in the CSS curve (Figure 3a), number of hits curve (Figure 3b), and *S_r_* curve (Figure 3e) were displayed at about 70 s. The *b*-value (Figure 3c) experienced a general decrease (increase in crack activity) and an increase in variations that also correlated to an increase in activities in CSS, number of hits, *S_r_*, and *H (t).* The first decreasing activity (first crack) detected in the *b*-value also occurred at about 70 s. To further corroborate the occurrence of the first crack, Figure 3d shows the AE activity of *H (t).* The first sudden increase in the *H (t)* curve (Figure 3d) also occurred at about 70 s.

After the occurrence of the micro-crack, there was an increase in all AE activities (CSS, number of hits, *S_r_*, *H (t)*) and a general decrease in *b*-value. This first crack detection using this analysis was similarly carried out in previous studies [22,50]. In addition, the first visible crack was detected during the test in the PRECC beam at about 100 s. In the load vs. time curve (Figure 3), the load was applied using a displacement control until the occurrence of the first visible crack. Both the load vs. time and AE activity vs. time curves showed very similar trends beyond the detection of the first crack. The same analysis on the aforementioned AE parameters was applied for all the other tested beams. Based on these results, the AE analysis proved its effectiveness in detecting the first crack and crack propagation until failure and coincided with the results obtained from the load vs. time curves beyond the first crack in all tested beams.

### 6.2. Time to First Crack Detection

**Figure 4:**
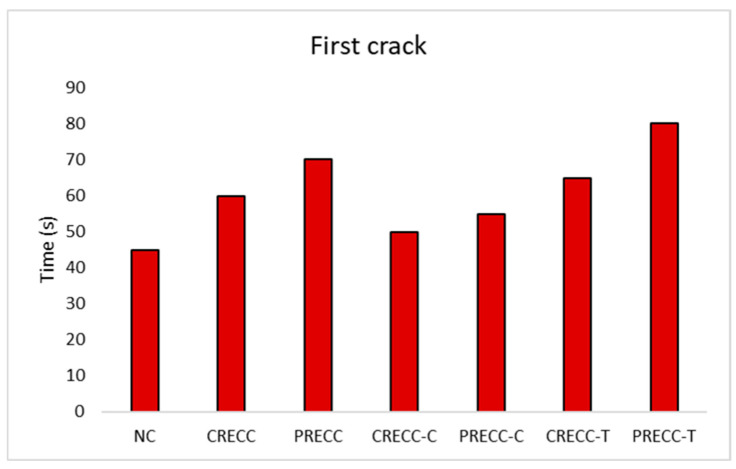
Time of the first crack load for all beams.

Figure 4 shows the time for the first crack for all tested beams. For the control beams (NC, CRECC, and PRECC), it is evident that the RECC beams showed a delayed first crack when compared to the NC beam (45 s). The CRECC beam displayed the first crack at about 60 s, while the PRECC beam showed the first crack at about 70 s. This could be due to the higher tensile strength of the RECC beams (Table 2). In terms of AE parameters, both CRECC and PRECC beams resulted in an average number of hits of 302 and 344 hits, average CSS values of 0.42 and 0.48 pV.s, and average *b*-values of 1.19 and 1.59, respectively, at the first crack detection (Table 5). On the other hand, the NC beam showed an average number of hits of 171.3, an average CSS value of 0.15 pV.s, and an average *b*-value of 2 (at the first crack). Referring to beams repaired in the compression zone (CRECC-C and PRECC-C), the time of the first crack in those beams was very comparable to the NC beam. CRECC-C and PRECC-C beams exhibited the first crack at 50 s and 55 s, respectively. The comparable time of the initiation of the first crack in beams repaired in the compression zone and the NC beam is related to the fact that the first crack occurred in the tension zone of the beam (bottom part), which is made with the NC mixture in both the fully cast NC beam or beams repaired in the compression zone. The slight increase in the time of the first crack in CRECC-C and PRECC-C beams compared to the NC beam (55 s compared to 50 s) is maybe due to the higher compression strain of CRECC-C and PRECC-C compared to NC [1]. With regard to beams repaired in the tension zone, both CRECC-T and PRECC-T showed a more delayed first crack (65 s and 80 s, respectively) due to the higher tensile strength (when compared to the NC beam) [1]. The CRECC-C beam was accompanied by an average number of hits of 251.3 hits, an average CSS value of 1.79 pV.s, and an average *b*-value of 1.53, while PRECC-C was accompanied by an average number of hits of 273.6 hits, an average CSS value of 3.3 pV.s, and an average *b*-value of 1.15 at the time of first crack (Table 5). In addition, both CREEC-T and PRECC-T were followed by an average of number of hits of 321.3 and 395.3 hits, average CSS values of 0.85 and 3.47 pV.s, and average *b*-values of 1.4 and 2.71, respectively, at the time of first crack (Table 5). AE analysis, therefore, has shown efficacy in obtaining the time for the first crack (first change in slope in AE parameter curves) for all tested beams.

### 6.3. Impact of Rubber Particle Size on the AE Parameters

As previously illustrated, this study included two different rubber particle sizes incorporated in RECCs: crumb rubber (CRECC) and powder rubber (PRECC). Two beams were fully cast in RECCs, and four other beams were repaired using RECCs in either the tension side or compression side of the beam. Table 5 shows the AE parameters at the first crack and ultimate load. It can be concluded that the rubber particle size utilized had a noticeable effect on the AE parameters. For instance, the NC beam displayed an average number of hits of 18,032 hits, average CSS of 70.13 pV.s, and an average *b*-value of 0.47 at the ultimate load, while CRECC and PRECC displayed an average number of hits of 19,163 and 19,625, an average CSS of 28.7 and 108.8 pV.s, and an average *b*-value of 0.64 and 0.82, respectively. In addition, beams repaired using powder rubber (PR) seemed to have a higher number of AE activities/parameters when compared to beams repaired in crumb rubber (CR).

The different types of rubber particles (CR or PR) used in the strengthening seemed to also have a distinguished effect on the AE parameters. For example, with reference to beams repaired in compression, CRECC-C displayed an average number of hits (at the ultimate load) of 18,702, an average CSS of 15.13, and an average *b*-value of 0.66, respectively, while PRECC-C exhibited an average number of hits of 19,025, an average CSS value of 62.3, and an average *b*-value of 0.4 at the ultimate load, respectively. For beams repaired in tension, CRECC-T displayed an average number of hits at the ultimate load of 19,117, a CSS average of 59.13 pV.s, and average *b*-value of 0.08, while PRECC-T displayed an average number of hits of 16,854, average CSS of 61.7, and an average *b*-value of 0.38. It is noticeable that beams with CR in general displayed a lower number of hits and CSS (lower number of hits and CSS) than beams with PR (except PRECC-T). To further elaborate, the reasoning behind the lower strength accompanied by CR could be due to the larger particle size of the crumb rubber that reduced the strength of the rubber–mortar interface, which resulted in the propagation of micro-cracks, contributing to a weaker strength that limited the continuation of deformation beyond the yield point [1]. PR, on the other hand, is smaller in particle size and exhibits a stronger rubber–mortar interfacial strength.

Moreover, beams with CR or PR generally displayed higher ductility and strain capacity (higher number of hits) than that of the NC beam due to the presence of fibers, which are known to transfer the stress over the cracked section, resulting in a higher tensile strain capacity. Other reasons include the presence of rubber particles, which enhance the strain capacity and deformability of the RECC beams. It should be noted that the PRECC-T beam displayed the least average number of hits (when compared to all beams). This is attributed to the fact that all beams failed in flexural failure, while the PRECC-T beams experienced a failure between the NC–RECC interface (debonding).

### 6.4. Impact of Repair Layer/Sensor Location on AE Parameters

As shown in Figure 2, all tested beams included three attached sensors. One sensor (sensor 1) was attached at the top of the beams (compression zone), and two sensors (sensors 2 and 3) were attached at the bottom (tension zone) of the beams. In general, sensor 1 (placed in the compression zone) in beams repaired in the tension zone showed a lower number of AE activity (CSS, number of hits, and *b*-value) when compared to sensors 2 and 3 (placed in the tension zone) due to the lack of fibers and rubber in these beams (top part is made with NC mixture). The higher number of AE activities resulting from sensors 2 and 3 was due to the presence of fibers and rubber particles that both allow for higher deformation and, therefore, higher cracking and AE activities. For instance, in Table 5, CRECC-T displayed 16,384 hits in sensor 1, 22,166 in sensor 2, and 18,800 in sensor 3. PRECC-T also showed 14,445 hits in sensor 1, 19,542 in sensor 2, and 16,575 in sensor 3.

Furthermore, in beams repaired in compression, sensor 1 (compression zone) showed a lower number of activity than that of sensors 2 and 3 (tension zone) even though the repaired layer was in the compression zone. The lower number of AE activities displayed by sensor 1 is attributed to the fact that beams repaired in compression failed in flex, and, therefore, the highest number of cracks (highest number of activities) existed in the tension zone of the beam. For instance, CRECC-C showed 16,030, 21,685, and 18,391 hits for sensors 1, 2, and 3, respectively. Also, PRECC-C displayed 16,305, 22,060, and 18,709 hits for sensors 1, 2, and 3, respectively. Repair layer location (tension or compression side of the beam) resulted in a significant effect on the other studied AE parameters (similar to the above trends of the number of hits) and was further accentuated through the position of the sensor placement.

**Figure 5:**
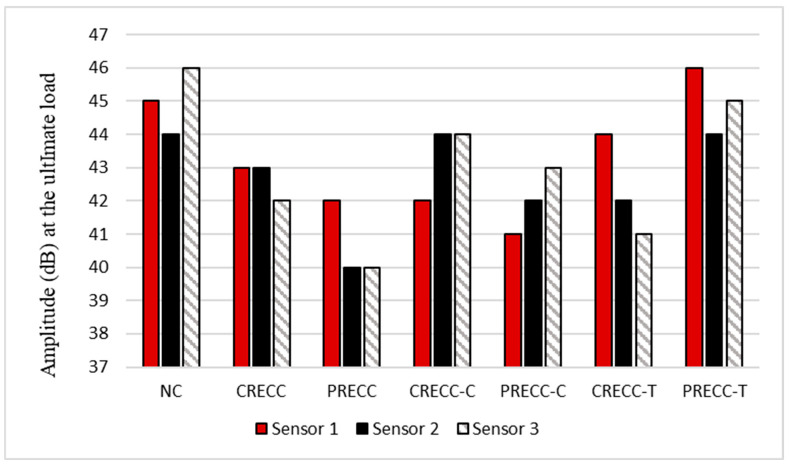
Variations in the signal amplitudes (ultimate load) of the three sensors for all beams.

To highlight the effect of multi-layers on AE signal characteristics, the values of the signal amplitude recorded from the three sensors were evaluated. Referring to Figure 5, it is evident that there are some variations in the values of amplitude (at the ultimate load stage) among the three sensors. Yet, in fully cast beams (NC, PRECC, and CRECC), the values of the amplitude were very close in the three sensors. For the NC beam, sensors 1, 2, and 3 at the ultimate load displayed amplitude values of 45, 44, and 46 dB, respectively. The CRECC beam showed amplitude values of 43, 43, and 42 dB for sensors 1, 2, and 3, respectively. For sensor 1 at the ultimate load in the PRECC beam, an amplitude value of 42 dB was displayed, while sensors 2 and 3 at the ultimate load displayed amplitudes of 40 and 40 dB, respectively. The closeness of the amplitude values is due to the homogeneousness of the materials, thus yielding similar wave propagation characteristics. On the other hand, repaired beams either in compression or tension showed noticeable variations in the three sensors. For instance, in the PRECC-T beam, the value of the amplitude at the ultimate load for sensor 1 was 46, while the values for the amplitudes at the ultimate load for sensors 2 and 3 (tension zone) were 44 and 45, respectively.

The variations in the values of the amplitudes are due to the difference in the materials of the two layers. This phenomenon is known as signal attenuation, which tends to occur when there is difference in densities between two materials, as well as lack of homogeneousness, which in this study occurred in repaired beams [51,52]. The type of concrete used in the repair layers also proved to be another factor that affected the values of the amplitude. Rubber is known to have sound absorbing characteristics, which tend to lower the value of the amplitude [21]. It is evident that in all repaired beams the NC layer displayed a higher amplitude, whether it was placed in the compression or tension zone of the beam. For instance, in the CRECC-C beam, an amplitude value of 42 dB was shown in sensor 1, while amplitude values of 44 and 44 dB were shown in sensors 2 and 3, respectively.

### 6.5. Damage Classification by AE Intensity Analysis

**Figure 6:**
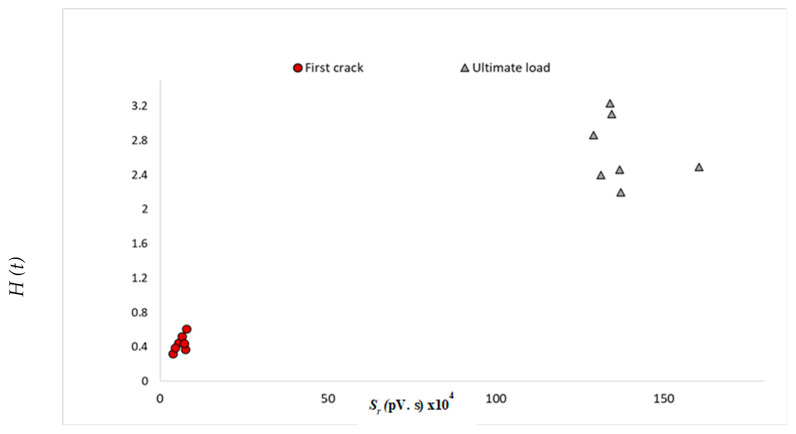
Crack classification chart for all tested beams.

As shown in the previous sections, AE analysis proved to be useful in detecting different damage stages and was beneficial in understanding the effect of the repair material used. To categorize the type of damage that occurred, intensity analysis parameters (*H (t)* and *S_r_*) were correlated to two stages of cracking (first crack and ultimate load). The values of *H (t)* and *S_r_* acquired from all the three sensors are illustrated in Table 5. The average values for the three sensors of *H (t)* and *S_r_* (for the seven tested beams) were calculated and then placed in Table 6 to create the chart, shown in Figure 6. This chart is used to represent the progression of cracking stages at both the first crack and ultimate load for all the tested beams. This chart included two ranges for *H (t)*, as well as *S_r_*. For *H (t);* the values ranged from 0.32 to 0.61 at the first crack and 2.2 to 3.23 for cracks at the ultimate load, respectively. Furthermore, for *S_r_*, the magnitudes varied from 3.8 to 7.8 × 10^4^ pV.s at the first crack and 129.1 to 160 × 10^4^ pV.s, correlating to the progression of cracks at the ultimate load, respectively. Both the values of *H (t)* and *S_r_* can be employed to represent the stages of cracking. For instance, an *H (t)* value of 2.82 and an *S_r_* value of 129 × 10^4^ pV.s. represent the stage of cracking at the ultimate load (as shown in Figure 6). This chart can possibly be used as a damage diagnosis tool for composite beams incorporating RECCs. Similar charts have been executed successfully in different investigations and were utilized in damage quantification of reinforced concrete structures [53,54,55].

## 7. Conclusions

This study utilized AE monitoring to analyze the crack initiation and propagation process of seven large-scale beams. Flexural tests were performed on three control beams (NC, CRECC, and PRECC) and four other NC beams that were either repaired with an RECC layer in the compression or tension zone of the beam. The RECC layers contained PVA and two types of recycled rubber with different particle sizes (4.75 and 0.4 mm). Several AE analyses were performed, and the following conclusions were drawn:

- AE parameters such as number of hits, CSS, *b*-value, *H (t),* and *S_r_* were found to be useful in understanding the cracking behavior of all tested beams, including the multi-layered beams. The number of hits, CSS, and *S_r_* collected during the loading period displayed an overall increase until the ultimate load. The overall increase was an indication of the crack initiation and propagation until failure. *b*-value, in contrast, experienced an overall decrease until the ultimate load. *H (t)* showed jumps and fluctuations that correlated to AE changes in slopes, displayed in the number of hits, CSS, *b*-value, and *S_r_* curves.
- The time for the first crack of the beam was experimentally detected and successfully confirmed through the analysis of the number of hits, CSS, *b*-value, *H (t),* and *S_r_*. The first crack was spotted at the first change of slope in the CSS, number of hits, and *S_r_* curves. For the *b*-value, the first crack was noticed at the first significant decreasing activity. The *H (t)* curve also showed the first crack at the first sudden activity.
- The inclusion of rubber in concrete mixtures seemed to have an impact on AE parameters such as number of hits. It was found that beams with rubber particles (RECC beams) showed higher AE activities compared to beams without rubber (NC beam). In addition, the use of smaller rubber size (PR) showed a higher number of hits when compared to beams with a larger rubber size (CR).
- The region with the highest cracking activity in the beam was found to have the highest impact on AE activities, regardless of the repair layer location. For example, when the repair layer was placed in the tension zone (NC layer at the top and RECC layer at the bottom), sensors 2 and 3, which were placed in the tension zone (highest cracking activities), displayed the highest number of AE events (compared to sensor 1, placed on the top layer). Also, when the repair layer was placed in the compression zone (NC layer at the bottom and RECC layer at the top), sensors 2 and 3 (placed at the bottom) still displayed the highest number of AE events due to the highest cracking activities at the bottom side of the beam.
- Analyzing the amplitude values revealed a wave attenuation in beams with multi-layers compared to the single layer beams (fully cast beams). It was found that in fully cast beams (NC, CRECC, or PRECC), the values of the amplitude from the three sensors were very close, while in repaired beams (either in compression or tension) the amplitude experienced some signal attenuation. This is owing to (a) the presence of rubber in the repair material (due to its sound absorbing capacity) and (b) due to the presence of two non-homogenous materials with two different densities (NC and RECCs).
- Intensity analysis was utilized to develop a damage quantification chart. The two intensity analysis parameters, *H (t)* and *S_r_*, were utilized to represent two cracking stages: first crack and ultimate load. For each parameter, there was a range of numbers representing a crack quantification stage. The chart can be used as a tool to categorize and quantify damage severity in terms of crack growth in NC–RECC composite beams.

## Figures and Tables

**Table 1:** Mix design for all tested beams [1].

Mixture No.	Mixture ID	BC	C/BC	SCM (type)	SCM/ BC	S/BC	C.A./BC	W/BC	PVA (Volume%)	CR/SS (Volume%)	PR/SS	F′_c_ (MPa)	STS (MPa)	Modulus of Elasticity (GPa)
1	NC	1	1	-	-	1.52	1.82	0.4	-	-	-	59	4.4	22
2	CRECC	1	0.45	FA	0.55	0.29	-	0.27	2	0.2	-	59.6	6.5	17
3	PRECC	1	0.45	FA	0.55	0.29	-	0.27	2	-	0.2	64.2	8.6	18.2

**Table 2:** Flexural test results for all tested beams [1].

Beam #	Beam ID	Load Capacity (kN)			Failure Mode	Cracking at Failure Stage	
		First Crack	Ultimate	Yield		Number	Maximum Width (mm)
B1	NC	25.2	390.5	6.8	Flexure	23	3.2
B2	CRECC	29.9	414.9	6.4	Flexure	39	2
B3	PRECC	35.6	425.4	5.1	Flexure	36	2.2
B4	NC-CRECC-C	25.5	404.1	8.5	Flexure	32	3.6
B5	NC-PRECC-C	26.8	410.4	8.2	Flexure	28	4.2
B6	NC-CRECC-T	32.8	411.9	6.5	Flexure	30	1.5
B7	NC-PRECC-T	36.9	368.3	-	Debonding	21	0.9

**Table 3:** Pre-testing AE data built-in filter ranges.

AE Data Setup	
Threshold	40 dB_AE_
Sample rate	1 MSPS
Pre-trigger	256 µs
Length	1 k points
Preamp gain	40 dB
Peak definition	200 µs
Hit definition	800 µs
Hit lockout time	1000 µs
Maximum duration	1000 µs

**Table 4:** Post-testing AE data amplitude-duration filter ranges.

Amplitude (dB)	Duration (µs)	
	Lower	Upper
40 ≤ A < 45	0	400
45 ≤ A < 48	0	500
48 ≤ A < 52	0	600
52 ≤ A < 56	0	700
56 ≤ A < 60	100	800
60 ≤ A < 65	300	1000
65 ≤ A < 70	500	2000
70 ≤ A < 80	1000	4000
80 ≤ A < 90	2000	7000
90 ≤ A < 100	3000	10,000

**Table 5:** AE parameters obtained at the first crack and ultimate load stages.

Beam Number	Sensor	Amplitude		CSS × 10^7^		*b*-Value		Number of Hits		*S_r_* × 10^4^		*H (t)*	
		First Crack	Ultimate Load	First Crack	Ultimate Load	First Crack	Ultimate Load	First Crack	Ultimate Load	First Crack	Ultimate Load	First Crack	Ultimate Load
NC	CH-1	48	45	0.12	57.2	2.11	0.58	113	15,454	2.66	123.5	0.23	2.3
	CH-2	46	44	0.19	87.8	1.83	0.35	244	20,910	4.65	155.6	0.45	2.1
	CH-3	47	46	0.14	65.4	2.08	0.49	157	17,733	4.09	134	0.26	2.15
CRECC	CH-1	42	43	0.36	22.5	1.70	0.82	260	16,424	4.69	131.5	0.36	3.08
	CH-2	40	43	0.48	32.4	0.80	0.42	343	22,220	6.35	140	0.51	2.21
	CH-3	40	42	0.42	31.4	1.07	0.68	303	18,845	5.21	122.4	0.47	2.01
PRECC	CH-1	42	42	0.41	76.8	1.95	0.92	295	16,820	5.73	120	0.45	3.68
	CH-2	41	40	0.55	154	1.29	0.68	391	22,755	7.32	150	0.57	2.87
	CH-3	41	40	0.47	95.6	1.55	0.86	345	19,300	6.29	132	0.53	3.15
CRECC-C	CH-1	40	42	0.97	6.79	1.92	0.72	216	16,030	3.89	132.3	0.35	2.75
	CH-2	44	44	2.80	24.5	1.01	0.59	290	21,685	5.33	139.6	0.42	2.16
	CH-3	43	44	1.61	14.1	1.68	0.66	248	18,391	4.36	138.5	0.39	2.41
PRECC-C	CH-1	40	41	2.05	51.8	1.76	0.62	236	16,305	6.79	132.3	0.30	2.82
	CH-2	43	42	4.04	72.7	0.83	0.24	309	22,060	8.33	125.5	0.44	3.60
	CH-3	44	43	3.81	62.4	0.85	0.34	276	18,709	7.46	129.5	0.38	2.06
CRECC-T	CH-1	44	44	0.78	45.5	1.66	0.1	275	16,384	6.34	161	0.23	3.53
	CH-2	40	42	0.92	81	1.26	0.06	367	22,166	8.21	155	0.75	2.82
	CH-3	41	41	0.84	50.9	1.29	0.08	322	18,800	7.15	134	0.33	2.98
PRECC-T	CH-1	45	46	3.02	43.9	2.72	0.45	340	14,445	6.83	143	0.54	2.40
	CH-2	40	44	3.87	81.5	2.80	0.31	450	19,542	8.87	174	0.68	3.12
	CH-3	40	45	3.52	59.7	2.60	0.40	396	16,575	7.71	163	0.60	1.95

**Table 6:** Severity analysis parameters obtained at two different cracking stages.

Beam ID	Avg *S_r_* (pV.s) × 10^4^		Avg *H (t)*	
	First Crack	Ultimate Load	First Crack	Ultimate Load
NC	3.8	137.7	0.32	2.2
CRECC	5.42	131.3	0.45	2.4
PRECC	6.45	134	0.52	3.23
CRECC-C	4.52	136.8	0.39	2.46
PRECC-C	7.53	129.1	0.37	2.82
CRECC-T	7.23	134.6	0.44	3.11
PRECC-T	7.80	160	0.61	2.49

## Data Availability

All data are included in the main body of the paper.
